# THE PREVALENCE OF HLA DQ2 AND DQ8 IN PATIENTS WITH CELIAC DISEASE, IN FAMILY AND IN GENERAL POPULATION

**DOI:** 10.1590/S0102-67202015000300009

**Published:** 2015

**Authors:** Lucila Arantes CECILIO, Mauro W. BONATTO

**Affiliations:** 1 Gastroclinic Clinic; 2 School of Medicine, Faculdade Assis Gurgacz - FAG, Cascavel, PR, Brazil

**Keywords:** Celiac disease, HLA Antigens

## Abstract

**Background:**

Celiac disease is an enteropathy characterized by gluten sensitivity and broad clinical aspect. Has a multifactorial cause and depends on genetic, immunological and environmental factors for its development. The genetic influence is given mostly by the human leukocyte antigens HLA DQ2 and DQ8.

**Aim:**

To evaluate the prevalence of human leukocyte antigens DQ2 and DQ8 in three different groups: patients with celiac disease, first-degree relatives and the general population.

**Method:**

Retrospective analysis that evaluated serologic and endoscopic data of 74 patients with celiac disease and 109 non-celiac, which were subdivided into two subgroups: non-celiac who had first-degree relatives with celiac and non-celiac who did not. All patients underwent laboratory examination for screening genetic sensitivity given by HLA DQ2 and HLA DQ8 by.

**Results:**

The presence of HLA DQ2 and DQ8 was identified in 98,4% of 74 celiac patients, of which 79,7% had only HLA DQ2; 8,1% had only HLA DQ8 and 10,8% had both antigens histocompatibility. In the group of relatives of celiac patients, were included 29 patients; among them, 89,6% had HLA DQ2 and/or DQ8; 76% only the HLA DQ2, 10,3% only HLA DQ8 and 3,4% presented both human leukocyte antigens (HLA).

**Conclusion:**

HLA DQ2/DQ8 was present in 98,4% of celiac patients; 89,6% relatives of celiac family and in 55,4% of people from the general population without family celiac.

## Abstract (pt)

**Racional:**

A doença celíaca é síndrome disabsortiva, autoimune, caracterizada pela sensibilidade ao glúten. Apresenta quadro clínico amplo e causa multifatorial, incluindo fatores genéticos, imunológicos e ambientais. O fator genético é dado, em sua maioria, pelo antígeno de histocompatibilidade HLA DQ2 e DQ8.

**Objetivo:**

Avaliar a prevalência do HLA DQ2 e DQ8 em três diferentes grupos: portadores de doença celíaca, em seus familiares de primeiro grau e na população geral.

**Método:**

Análise retrospectiva a partir de um banco de dados informatizado onde foram avaliados dados sorológicos de 74 pacientes portadores de doença celíaca e 109 não celíacos. Os não celíacos foram subdivididos em dois subgrupos: os que possuíam familiares de primeiro grau com doença celíaca e os que não. Todos foram submetidos à pesquisa de sensibilidade genética dada pelo HLA DQ2 e pelo HLA DQ8.

**Resultados:**

A presença do HLA DQ2 e DQ8 foi identificada em 98,4% dos 74 pacientes celíacos; destes, 79,7% apresentavam apenas HLA DQ2; 8,1% apenas HLA DQ8 e 10,8% os dois antígenos de histocompatibilidade. No grupo dos familiares de celíacos, foram avaliados 29 pacientes, dentre os quais 89,6% apresentavam o HLA DQ2 e/ou DQ8; destes, 76% apenas o HLA DQ2, 10,3% apenas o HLA DQ8 e 3,4% apresentou os dois antígenos de histocompatibilidade. Na população geral sem familiares celíacos, foram avaliados 80 pacientes; dentre eles, 53,7% apresentaram o antígeno, sendo 41,2% apenas o HLA DQ2, 11,3% apenas o HLA DQ8 e 1,2% tanto o HLA DQ2 quanto o HLA DQ8.

**Conclusão:**

O alelo HLA DQ2/DQ8 se fez presente em 98,4% dos pacientes celíacos; 89,6% dos familiares de celíacos; e em 55,4% das pessoas da população geral sem familiares celíacos.

## INTRODUCTION

Also known as "gluten intolerance", "non-tropical sprue" or "gluten-induced enteropathy," celiac disease (CD) is an autoimmune enteropathy framed within disabsortive syndromes. The disease is permanent, and only occurs in genetically predisposed patients, when they are exposed to dietary gluten. The disease involves various mechanisms in its pathogenesis and has a range of clinical presentation, so it is necessary to maintain a low threshold for diagnostic suspicion. Despite these features, the CD basically depends on three main aspects to become active: the ingestion of gluten - etiologic agent of the disease -, dysfunction of intestinal mucosal barrier and genetic predisposition (holotypes HLA DQ2 / DQ8).

Gluten represents a mixture of proteins found in the endosperm of cereal seeds (wheat, rye, barley, oats, etc.). And, among its components, the main one is gliadin, which is the toxic fraction and is directly involved in the pathogenesis of CD.

The gliadin meets the transglutaminase tissue (TGt) in the intestinal lumen, thus forming a macromolecular complex which can be recognized as antigens by antigen presenting cells via allele of the major histocompatibility complex class II, namely HLA-DQ2 and HLA-DQ8.

Based on the factors that trigger the disease previously described, and knowing the importance of genetic markers HLA-DQ2 and HLA-DQ8 in the pathophysiology of the disease, we began collecting data from a large retrospective study to evaluate the incidence of these genetic markers in the West of Paraná State, Brazil, population, location where the ingestion of gluten, cultural habits and the genetically predisposed population represent a high level.

Knowing that the disease predominantly affects white individuals of Caucasian origin, and evaluating the ethnic formation of the State, which is given predominantly Caucasian descent, the objective of the research covers the comparison between the data reported in the literature with data collected from patients of West of Paraná population, especially with regard to the high prevalence of genetic HLA antigens in the general population (20-30%).

## METHODS

### Population

Patients admitted to Gastroclínica Cascavel in the period from January 1, 2008 until May 14, 2012 who underwent endoscopic and serological tests. The study excluded patients who did not present the results of serological tests and/or patients with incorrect or insufficient data.

### Database

Retrospectively were reviewed the records of 183 patients contained in a computerized database containing personal, endoscopic, serological records and other patient information. Were selected personal information and serological features that made reference to antigen histocompatibility. All patients were informed about the prospective insertion of results in a database for use for clinical research.

### Statistical analysis

Data were analyzed using descriptive statistics: arithmetic mean, standard deviation, frequency and percentage.

## RESULTS

During the four years included on this study, were analyzed 414 medical records of patients who may be unrolled in the study. Then, were selected serological results that informed the presence and/or absence of HLA DQ2 and DQ8, and other features that could bring additional information to the study, for example, the degree of kinship between some of the patients.

**FIGURE 1:**
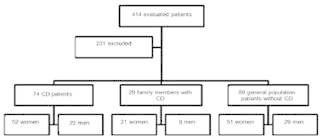
Study design with the evaluated population

Among the 414 records, 263 were from patients with CD, but 189 were excluded for not having all the inclusion criteria. The other 151 selected records correspond to the general population of patients who did not present CD, which were divided in two subgroups: the first included patients from the general population without CD, but with first-degree relatives with celiac disease (32) and the second subgroup patients that did not present neither CD nor first-degree relatives with CD (119). Still, excluding those who did not fit the inclusion criteria, remained 29 patients evaluated in the first subgroup and 80 patients in the second subgroup ( Figure 1 ).

### Serological results

**FIGURE 2:**
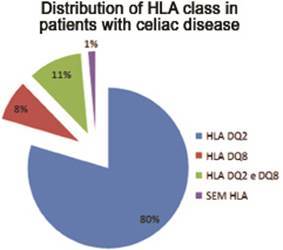
Characteristics of surface antigens in celiac disease

In the first evaluated group - with DC - 73 (98.6%) patients had the histocompatibility antigen, and 59 (79.7%) with only the HLA DQ2, six (8.1%) only with the HLA DQ8 - eight (10.8%) with both surface markers ( Figure 2 ).

**FIGURE 3:**
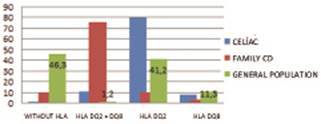
Prevalence of the different HLA class in three groups (%)

 Among non celiac patients but with first degree celiac relatives, 26 (89.7%) had the HLA markers, 22 (76%) with only the HLA DQ2; three (10.3%) with HLA-DQ8 and one (3.4%) with both HLA-DQ2 and HLA DQ8. In the subgroup of non-celiac and without celiac families, 43 patients (53.7%) were HLA being 33 (41.2%) only with HLA DQ2; nine (11.3%) HLA-DQ8, and only one (1.2%) with both ( Figure 3 ).

## DISCUSSION

Celiac disease is self immune disease whose causative environmental agent is gluten, present in barley, rye, oats and wheat. It has a strong genetic influence and variations in their phenotype (according to the stage at which the disease is found); therefore requires high degree of suspicion by the observer.

Three factors are involved in the pathogenesis of the disease: ingestion of gluten; changes in the junctions of the intestinal mucosa - gliadin able to cross the barrier and to activate the inflammatory process -, and the presence of the genetic factor, given by some specific HLA.

The humoral nature, hereditary and polygenic CD has great influence in triggering the disease. Genetic factors, given by surface markers HLA DQ2 and HLA DQ8 are found in high levels in the general population.

In celiac patients with active and present such disease markers, gluten interacts with HLA causing abnormal immune response in the intestinal mucosa and tissue injury.

Among the world's population, HLA typing is present in 98.6% of patients with CD, with a high negative predictive value. Moreover, in the general population who do not have the diagnosis of CD, HLA-DQ2 and/or HLA-DQ8 is present in approximately 40% of the population, and this percentage increases among patients who are not celiac but has first-degree relatives with celiac disease.

It is worth remembering that HLA is a genetic trait, and therefore its presence in the general population has a higher prevalence in relatives of celiac patients. The closer are the relatives, more prevalent may be antigen histocompatibility.

Despite the absence of specific HLA as negative predictive value for the development of the disease, they do not act as diagnostic criteria for confirmation, since they are also present with high prevalence in the general population. In this study, it was possible to report the high prevalence of these antigens in western Paraná, Brazil, both in celiac as in non-celiac.

Were analyzed some features of the described population, its genetic heterogeneity, ethnics, environmental and cultural influences in order to clarify the reason for the higher prevalence of such antigens in this region compared to other places in the literature.

In relationship to Paraná ethnicity, it was observed that the colonization of Western region occurred by 1930, with the wood cycle, and was given predominantly by Italian and German. The culture of these ethnicities and eating habits today prevails in this region, which can be observed from high gluten ingestion. According to IBGE data, the ethnicity of the population of Cascavel is at 70.15% made up of white, 26.25% for brown, 2.59% for black and 0.88% yellow. Currently within the white race the highest percentage is still given by Italian and German. It is known, according to data from the literature, that celiac disease affects predominantly white individuals of Caucasian origin at the rate of one for every hundred individuals.

Based on such information, it is seen that the predominant population in this region has characteristics that prevail habits and the onset of the disease and/or the high incidence of such genes.

 Moreover, it is noteworthy that there was compatibility between the data and findings in the patients celiac and other countries in regard to the characteristics and distribution of antigen histocompatibility.

It is reported, finally, the high prevalence worldwide and in Paraná HLA DQ2 and DQ8 markers, which varies from 20-40% of the population, without this meaning the onset of celiac disease.

## CONCLUSION

HLA DQ2/DQ8 was present in 98,4% of celiac patients; 89,6% relatives of celiac family and in 55,4% of people from the general population without family celiac.
